# JMIR Perioperative Medicine: A Global Journal for Publishing Interdisciplinary Innovations, Research, and Perspectives

**DOI:** 10.2196/54344

**Published:** 2023-11-21

**Authors:** Nidhi Rohatgi

**Affiliations:** 1 Stanford University School of Medicine Stanford, CA United States

**Keywords:** JMIR Perioperative Medicine, innovation, technology, digital health, research, interdisciplinary, perioperative medicine

## Abstract

*JMIR Perioperative Medicine* supports the dissemination of technological and data science–driven innovative research conducted by interdisciplinary teams in perioperative medicine. We invite contributions on a broad range of topics from clinicians, scientists, and allied health professionals from across the globe.

## Perioperative Medicine: From Opium to Artificial Intelligence

It was not until 1846 that the possibility of undergoing a major surgical procedure without experiencing excruciating pain was recorded [1]; this surgery was performed in the United States. Before this, it was opium, mandrake, alcohol, or simply “biting the bullet” for invasive procedures. This was also the first record of the use of technology in the operating theater with the introduction of the first “anesthetic machine” [2]. A few decades later, a Scottish surgeon introduced the concept of orotracheal intubation, and a century later, the modern ventilator was developed in Sweden [3]. All these discoveries and innovations led to seismic shifts in the field of perioperative medicine. As surgical and anesthetic techniques evolved, focus on systematic perioperative data recording and patient monitoring, outcomes, and experiences also emerged. In 1949, the idea of preoperative clinics was introduced in England “so the patients can arrive in the operating theater as strong and healthy as possible” [4]. In 2007, the World Health Organization created a surgical safety checklist that decreased surgical morbidity and mortality across 8 hospitals in different countries, and now, over 75% of institutions across 94 countries use these checklists [5]. In 2020, amid the COVID-19 pandemic, as surgeries came close to a standstill, exemplar collaborations across multiple high- and low-income countries occurred to study surgical safety in patients with COVID-19 [6]. Time and time again, the publication of an “aha moment,” an incredible breakthrough, in one corner of the world catalyzed a change in the care of surgical patients and the practice of perioperative medicine across the globe.

Today, technological advancements and digital innovations continue to transform perioperative medicine at an unprecedented pace compared to yesteryears. This field has become much more interdisciplinary than ever before. There has been an increasing amount of research to predict, prevent, or understand the short- and long-term impacts of physiological perturbances during the perioperative period on different organ systems. mHealth (mobile health) technologies are being used to educate surgical patients, caregivers, and clinicians about perioperative pain management or to collect patient health data, perioperative outcomes, or experiences. Resource utilization, access, and patient and clinician experience are being optimized through telemedicine and digital communication strategies [7]. Big data is being leveraged to drive quality improvement and research in some centers while others are driving innovations in the midst of paper charts [8]. Biases in the use of common devices such as pulse oximeters are being recognized in North America while some countries continue to struggle to get access to these common devices [9]. Artificial intelligence and machine learning, which seemed quixotic years ago, have made an unexpectedly large leap with several potential applications, such as reducing medication errors, perioperative risk assessment, early detection of clinical deterioration, predicting postoperative morbidity and mortality, streamlining workflows, creating personalized care pathways, real-time image analysis, providing clinical decision support, analysis of structured and unstructured data in electronic health records, or understanding health disparities [10-12]. Experts in perioperative medicine are investigating a broad range of topics—from sustainability and the carbon footprint of surgeries to taking a deeper dive into precision medicine using omics and biomarkers [13]. Some of these discoveries and research will reduce inequities and improve surgical safety on a global scale while others may improve outcomes and care delivery locally, both of which are important.

## JMIR Perioperative Medicine: Focus and Scope

*JMIR Perioperative Medicine* is a global, peer-reviewed, open access journal indexed in PubMed, PubMed Central, Directory of Open Access Journals (DOAJ Seal), EBSCO/EBSCO Essentials, and Sherpa/Romeo. This journal welcomes contributions from a diverse group of investigators and clinicians from across the globe. We accept clinical trials, meta-analyses, reviews, observational studies, quality improvement studies, research letters, viewpoints, and tutorials. We are committed to disseminating research and advancements in all aspects of perioperative medicine. This includes novel and promising formative studies that may inform future research in the field. We accept articles on a broad range of topics in perioperative medicine such as digital and technological innovations; patient monitoring; patient and clinician education; operating room management; anesthetic techniques; pain management; interdisciplinary care; perioperative risk assessment; blood management; best practices; access to surgery and critical care; quality; safety; cost-effectiveness; diversity, equity, and inclusion; climate change and sustainability; and global surgical initiatives.

Although substantial progress has been made in perioperative medicine, much remains to be done and new frontiers are yet to be explored. Life expectancy has increased and older patients with many more comorbidities are opting to undergo surgical procedures. Globally, 4.2 million patients still die within 30 days of surgery, with over half of these deaths occurring in low- and middle-income countries [14]. This amounts to 7.7% of deaths globally, making postoperative deaths the third leading contributor to mortality in the world after ischemic heart disease and stroke. This is a staggering statistic. There remain several unresolved clinical conundrums in perioperative medicine. Health care delivery is also tasked with creating innovative solutions to tackle workflow optimization, cost-effectiveness, emergency preparedness, workforce wellness, education, and communication, all while achieving the highest-quality patient outcomes and experiences. Perioperative medicine continues to face an attractive challenge, one that motivates all of us to think creatively, share and publish our knowledge and experiences, and build on each other’s ideas and innovations from across the world. *JMIR Perioperative Medicine* aims to be at the forefront of this movement for sharing interdisciplinary knowledge and innovation in perioperative medicine on a global scale. With our talented editorial board and editorial team, we provide our authors with the best experience from submission to post publication. We look forward to receiving inspiring and exciting submissions to this journal as we, in symbiosis, shape the field of perioperative medicine.

## Abbreviations

mHealth: mobile health

## References

[1] Jackson C, Morton W (1846). Improvement in surgical operations. US patent 4848.

[2] Macewen W (1880). General observations on the introduction of tracheal tubes by the mouth, instead of performing tracheotomy or laryngotomy. Br Med J.

[3] Engström C (1953). Respirator designed according to a new principle. Sven Lakartidn.

[4] Lee AJ (1949). The anaesthetic out-patient clinic. Anaesthesia.

[5] Delisle M, Pradarelli JC, Panda N, Koritsanszky L, Sonnay Y, Lipsitz S, Pearse R, Harrison EM, Biccard B, Weiser TG, Haynes AB (2020). Variation in global uptake of the Surgical Safety Checklist. Br J Surg.

[6] COVIDSurg Collaborative (2020). Mortality and pulmonary complications in patients undergoing surgery with perioperative SARS-CoV-2 infection: an international cohort study. Lancet.

[7] Milne-Ives M, Leyden J, Maramba I, Chatterjee A, Meinert E (2022). The potential impacts of a digital preoperative assessment service on appointments, travel-related carbon dioxide emissions, and user experience: case study. JMIR Perioper Med.

[8] Cole JH, Highland KB, Hughey SB, O'Shea BJ, Hauert T, Goldman AH, Balazs GC, Booth GJ (2023). The association between borderline dysnatremia and perioperative morbidity and mortality: retrospective cohort study of the American College of Surgeons National Surgical Quality Improvement program database. JMIR Perioper Med.

[9] Sjoding MW, Dickson RP, Iwashyna TJ, Gay SE, Valley TS (2020). Racial bias in pulse oximetry measurement. N Engl J Med.

[10] Ye J (2023). Patient safety of perioperative medication through the lens of digital health and artificial intelligence. JMIR Perioper Med.

[11] Kendale S, Bishara A, Burns M, Solomon S, Corriere M, Mathis M (2023). Machine learning for the prediction of procedural case durations developed using a large multicenter database: algorithm development and validation study. JMIR AI.

[12] Chen P, Chen L, Lin Y, Li G, Lai F, Lu C, Yang C, Chen K, Lin T (2022). Predicting postoperative mortality with deep neural networks and natural language processing: model development and validation. JMIR Med Inform.

[13] Ramaswamy P, Shah A, Kothari R, Schloemerkemper N, Methangkool E, Aleck A, Shapiro A, Dayal R, Young C, Spinner J, Deibler C, Wang K, Robinowitz D, Gandhi S (2022). An accessible clinical decision support system to curtail anesthetic greenhouse gases in a large health network: implementation study. JMIR Perioper Med.

[14] Nepogodiev D, Martin J, Biccard B, Makupe A, Bhangu A, National Institute for Health Research Global Health Research Unit on Global Surgery (2019). Global burden of postoperative death. Lancet.

